# Unraveling Lyophilization and Redispersion Effects on Miktoarm Polymer-Based Nanoformulations

**DOI:** 10.3390/ijms262010015

**Published:** 2025-10-15

**Authors:** Samaneh Yousefi Adlsadabad, Gabriel Théberge-Julien, Fatima Fernanda Portillo Gutierrez, Ricardo Beltran Medina, Ximena Matias Mercado, Éric Rhéaume, Jean-Claude Tardif, Ashok Kakkar

**Affiliations:** 1 Department of Chemistry, McGill University, 801 Sherbrooke Street West, Montreal, QC H3A 0B8, Canada; samaneh.yousefiadlsadabad@mail.mcgill.ca (S.Y.A.); a01367846@tec.mx (F.F.P.G.); ricardo.medina@mail.mcgill.ca (R.B.M.); ximena.matiasmercado@mail.mcgill.ca (X.M.M.); 2 Research Centre, Montreal Heart Institute, 5000 Belanger Street, Montreal, QC H1T 1C8, Canada; gabriel.theberge-julien@icm-mhi.org (G.T.-J.); eric.rheaume@icm-mhi.org (É.R.); jean-claude.tardif@icm-mhi.org (J.-C.T.); 3 Department of Medicine, Universite de Montreal, Montreal, QC H3T 1J4, Canada

**Keywords:** polymeric nanoparticles, lyophilization/redispersion, drug delivery, nanoformulations, curcumin-loaded nanoformulations

## Abstract

To enhance the scope of therapeutic interventions using star polymeric nanoparticles of desired concentrations, an understanding of the effect of converting aqueous formulations into stable redispersible dry powders by freeze drying on their physicochemical and biological properties is essential. We demonstrate that parameters such as the choice of the cryoprotectant, its molecular weight, and concentration play an important role during lyophilization and reconstitution processes. We hypothesized that utilizing cryoprotectants akin to shell-forming polymers may be ideal in protection against aggregation and keeping the nanostructures intact during lyophilization and reconstitution, as well as retaining the overall biological efficacy of their cargo. Through an evaluation of miktoarm polymer-based nanoparticles, we demonstrate that PEG_2k_ at 1% *w*/*v* concentration provides the optimized cryoprotection, and the resulting solid formulations upon redispersion in an aqueous medium preserve the desired nanoparticle and curcumin properties. PEG_2k_ at 1% *w*/*v* is more efficient than PEG_5k_ and saccharides including glucose, sucrose, trehalose, and mannitol in enhancing the integrity of micelles during lyophilization and reconstitution. Addition of PEG_2k_ 1% *w*/*v* (with or without lyophilization and redispersion) enhances drug release in PBS buffer, while it has no impact in the cell culture media. Nanoformulations protect endothelial cells from cytotoxicity of curcumin, and addition of cryoprotectant or the lyophilization/redispersion processes did not impair anti-inflammatory efficacy of curcumin.

## 1. Introduction

Diseases with a high morbidity rate, such as cardiovascular disorders and cancer, continue to pose significant health challenges. Despite considerable progress in the field of pharmacotherapy, the delivery of active pharmaceutical agents to desired sites remains challenging and a topical area of interest [1,2]. The poor pharmacokinetics properties of many drug candidates, such as their low water solubility, limited bioavailability, and stability, particularly among lipophilic drug candidates, necessitates the development of drug delivery systems to address these key issues [1,3,4]. Tremendous research has been dedicated to designing nanocarriers including polymeric [5], lipid-based, and inorganic nanoparticles to enhance drug delivery performance and overcome the pharmacokinetic limitations [6,7]. Polymeric micelles with their ease of preparation, surface modification, and better tunability, continue to attract significant interest for applications in nanomedicine [8]. Miktoarm polymers (*mikto*, Greek, meaning “different”) have demonstrated advantageous characteristics compared to their linear counterparts, including diversity in the tunability of the overall structure and ease of functionalization. The aqueous self-assemblies from these amphiphilic branched polymeric precursors exhibit higher colloidal stability, smaller and more uniform nanoparticles, denser corona, controlled cargo release, and multi-functionality originating from the presence of different functional arms in a single molecule [9]. We, along with other research groups, have leveraged this platform to develop a variety of soft nanoparticles and demonstrated their promising potential in drug delivery [10,11,12].

The long-term storage of aqueous nanoformulations remains a significant challenge, as drug leakage through diffusion or as a result of nanoparticle instability may alter their therapeutic efficacy [13]. In addition, the need to use large volumes of such formulations for dose-dependent therapies, restricts their usage for intravenous administration [14]. This necessitates the dehydration of nanoformulations and their subsequent redispersion into water at desired concentrations [15]. Freeze drying, often referred to as lyophilization, is an effective strategy for this purpose. This process involves low temperatures and pressure to remove water (ice crystals) from nanoparticles through a sublimation process [16]. In contrast to rotary evaporation and spray drying [17,18], which involve heat to remove solvents, the use of low temperatures/sublimation is more suitable to preserve drug properties. However, lyophilization presents its own challenges, including nanoparticle aggregation, structural disruption, and alterations in the size and morphology upon redispersion, which can negatively impact the stability and performance of the formulation [19]. The recent development of mRNA vaccines against COVID-19 have demonstrated the significance of having readily redispersible lyophilized nanoparticles [20]. Excipients acting as cryoprotectants are essential for preserving the original properties of nanoparticles following the freeze-drying process. Cryoprotectants are generally hydrophilic materials that form a glassy matrix around nanoparticles. The matrix functions as a pseudo-hydrating shell for nanoparticles, protecting them by reducing the mechanical stress during the freeze/drying process and preventing them from aggregation through the establishment of barrier between nanoparticles [21]. Various excipients have demonstrated cryoprotective properties, including sugars [22] (trehalose [23], sucrose [24,25], dextrose, glucose [26], mannitol [27], fructose, and sorbitol [28]), polymers (polyethylene glycol [29], polyvinyl alcohol, poloxamers [30], and polyvinylpyrrolidone [31]), and surfactants (tween 20). The choice of cryoprotectant, as well as its molecular weight and concentration, depends significantly on the type of lyophilizate [32], and it is essential to optimize these parameters [33,34]. Varied characteristics of cryoprotectants including their molecular composition, glass transition temperature, hydrogen bonding ability, interaction with the shell surface of nanoparticles, etc., can significantly influence their stabilization during lyophilization and redispersion. Based on the concentrations used during cryopreservation, excipients with high crystallinity could yield secondary mechanical forces on frozen nanoparticles, leading to aggregation. In addition, in nanocarriers with physically incorporated therapeutic cargo, the cryoprotectant should not interfere with drug release kinetics and their overall biological efficacy [35,36,37,38]. Numerous studies have explored the use of cryoprotectants to enhance the redistribution of various nanoparticle systems, including linear polymeric nanoparticles, silica nanoparticles [39,40], chitosan nanoparticles [41], nanoplexes [42], gold nanoparticles [43], nanocrystals [44], nanogels [45,46], and lipid nanoparticles [34,47,48].

An optimized cryoprotectant should not only preserve the morphology, surface charge, hydrodynamic diameter, and drug encapsulation efficiency of nanoparticles, but it should also help maintain the pharmacotherapeutic efficacy of the drug. Despite the inert characteristics of cryoprotectants, these can still influence nanoparticle–target site interaction and, in some cases, may induce toxicity [49]. These excipients can also alter drug release kinetics, as observed in insulin-loaded PLGA nanoparticles. The insulin release profile was evaluated using different cryoprotectants in comparison to nanoparticles without cryoprotectants, and trehalose was shown to increase the insulin release content, whereas sucrose, glucose, fructose, and sorbitol reduced it [50]. We were intrigued to utilize polyethylene glycol as a cryoprotectant due to its advantageous combination of properties. The latter include biocompatibility and the resemblance and affinity towards shell-forming polymers in nanoparticles, which might offer better protection against aggregation and help retain the nanoformulation integrity during freeze drying. Similar arguments suggest that it will also be an ideal candidate in retaining biological efficacy of the drug-loaded polymeric nanoparticles.

Studies associated with polymeric micelles are mostly limited to the physical stability of nanoparticles, and to the best of our knowledge, there have been limited studies on the lyophilization and redispersion of nanoparticles based on branched star polymers. Holzer et al. found that the dispersibility behavior of PLGA (poly lactic-co-glycolic acid)-based nanoparticles improved using sucrose and trehalose at concentrations of 1% and 2%, respectively [51]. In another study, Ayen et al. explored the lyophilization impacts and lyophilizate long-term storage on the physicochemical stability of doxorubicin-loaded PLA(PEG)_3_-based polymersomes. They found that 5% *w*/*v* inulin was a superior cryoprotectant helping to preserve the structural properties of the lyophilizate [52]. Cryoprotectants can also be integrated in situ into polymeric nanoformulations during the self-assembly process. Mihyar et al. incorporated sucrose as a cryoprotectant into mPEG-N-(2-benzoyloxypropyl) methacrylamide-based polymeric micelles during microfluidic self-assembly. The resulting nanoparticles exhibited 6-month stability at −20 °C [53]. Taking into account the considerable advantages of branched polymers compared to their linear counterparts, there is significant unmet need in determining the effect of lyophilization and redispersion of such nanoparticles [9].

In this study, we evaluated the effects of the lyophilization and redispersion of branched (AB_2_ [A = poly caprolactone (PCL_3.5k_), B = polyethylene glycol (PEG_2k_)])-based nanoformulations on their physical stability and biological properties. The evaluation of cryoprotectants compatible with the nanoformulation was conducted in two steps: (i) physical stability assessment through dynamic light scattering (DLS) and transmission electron microscopy (TEM) analyses and (ii) utilization of the effective least concentration of cryoprotectant for biological studies. Four different types of polysaccharides and polyethylene glycol with 2k and 5k molecular weights were explored for this purpose, and PEG_2k_, at a concentration of 1% *w*/*v*, demonstrated superior efficacy on the physical stability preservation. Using PEG as a compatible cryoprotectant for nanoformulations, we assessed the impact of the freeze-drying process on the nanoparticles’ morphology, drug release profile and kinetics, cytotoxicity, and the anti-inflammatory efficacy of curcumin-loaded nanoparticles. The selection of PEG as a cryoprotectant is based on its similarity to the hydrophilic shell of our miktoarm star nanoparticles and its suitability for maintaining the stability of relatively small polymeric nanoparticles. The drug release profile of curcumin-loaded nanoparticles with 1% PEG_2k_ in PBS media showed an enhanced release; while in cell culture media, the release profiles of the nanoparticles were almost the same with or without cryoprotectants. We demonstrate that an optimal concentration of polyethylene glycol as a cryoprotectant preserves the morphology and prevents aggregation upon redispersion after lyophilization. Its presence has no significant impact on cell viability and drug release parameters, and curcumin retains its anti-inflammatory activity post-processing.

## 2. Results and Discussion

### 2.1. Synthesis and Characterization of Miktoarm Polymer

The AB_2_-based miktoarm polymer utilized in this study was synthesized by adaptation of the procedure reported earlier by our group (Figure S1) [10], and the detailed synthetic procedure and polymer characterization using ^1^H-NMR (Figurs S2, S4 and S7–S9) ^13^C-NMR (Figures S3 and S5), MALDI-TOF (Figure S6), FT-IR (Figure S10), and GPC analysis (Figure S11) is provided in the Supplementary Material section. PEG was selected as the FDA-approved hydrophilic arm due to its biocompatibility and the ability to reduce the opsonization of nanoparticles and enhance the nanoparticle circulation time. PCL as a hydrophobic polyester offers biocompatibility and biodegradability. The star amphiphile, which consists of two PEG chains with a molecular weight of 2k each and a PCL arm of 3.5k, results in a hydrophilic fraction of 0.53. The latter is expected to lead to core–shell type micelles upon aqueous self-assembly [54].

### 2.2. Preparation of Nanoparticles and the Selection of an Optimal Cryoprotectant

**Figure 1:**
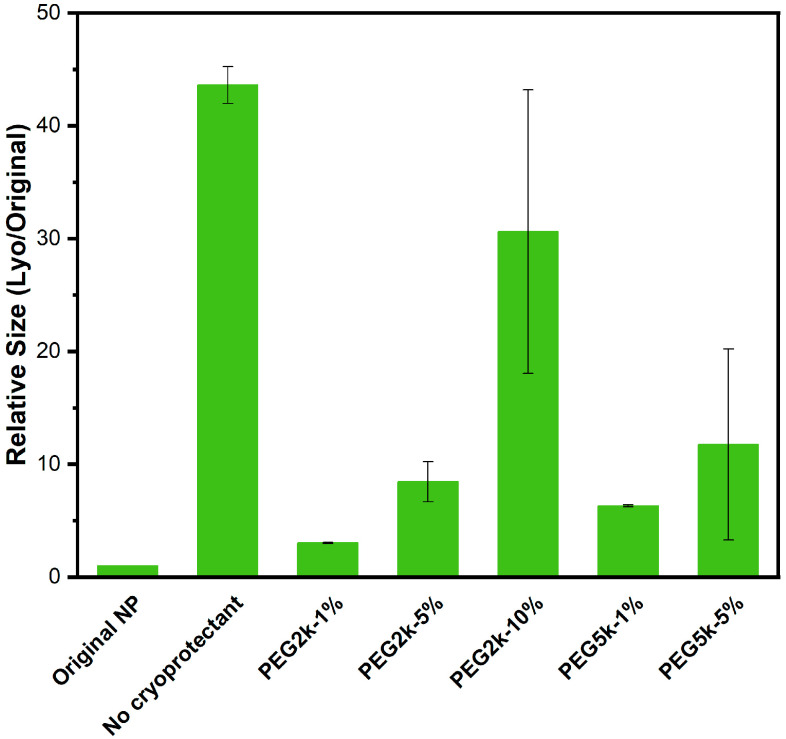
Cryoprotectant concentration assessment: Lyophilization with no cryoprotectant added, as well as with different concentrations (*w*/*v* %) of PEG_2k_ and PEG_5k_ as cryoprotectants were evaluated for blank nanoparticles. Relative hydrodynamic diameter (nm) of blank Lyo nanoparticles compared to original nanoparticles are reported. All measurements were carried out in triplicate. For nanoparticles containing PEG_2k_-10% and PEG_5k_-5%, we observed a very high percentage error and reported the best fit for measurements in duplicate.

Nanoparticles were prepared using co-solvent evaporation method, and their size and polydispersity were studied for the Original (no cryoprotectant added/non-lyophilized), NLyo (cryoprotectant added/non-lyophilized), and Lyo (cryoprotectant added/lyophilized nanoparticles). As expected, the lyophilized and then redispersed nanoparticles to which no cryoprotectant was added showed larger aggregates around 1677 ± 219 nm. We evaluated commonly utilized sugars such as glucose, trehalose, sucrose, and mannitol, as well as polyethylene glycol as cryoprotectants. Considering PEG_2k_ forms the integral hydrophilic component of our amphiphilic star polymers (AB_2_, B = PEG_2k_), we chose the cryoprotectant PEG molecular weight to be 2k or 5k. By taking into account the structural similarity of polyethylene glycol (PEG) to the nanoparticle shell forming composition of our miktoarm polymer precursors, we initially directed our studies primarily towards evaluating PEG as a cryoprotectant. PEG with molecular weight of 2kDa was tested at concentrations of 1%, 5%, and 10%, while PEG 5kDa was evaluated at 1% and 5% *w*/*v*. The relative diameters of the blank Lyo nanoparticles, compared to their original size with and without the addition of cryoprotectants, are summarized in Figure 1 and Table S1. The results showed that 1% *w*/*v* concentration of PEG is the optimal cryoprotectant level for nanoparticle suspension at 2.5 mg/mL. At this concentration, Lyo-nanoparticles containing PEG with both molecular weights demonstrated a smaller relative size compared to the original nanoparticles. Higher cryoprotectant concentrations resulted in larger relative sizes of redispersed nanoparticles with increased batch-to-batch variability due to the aggregation of excess cryoprotectants. 

**Figure 2:**
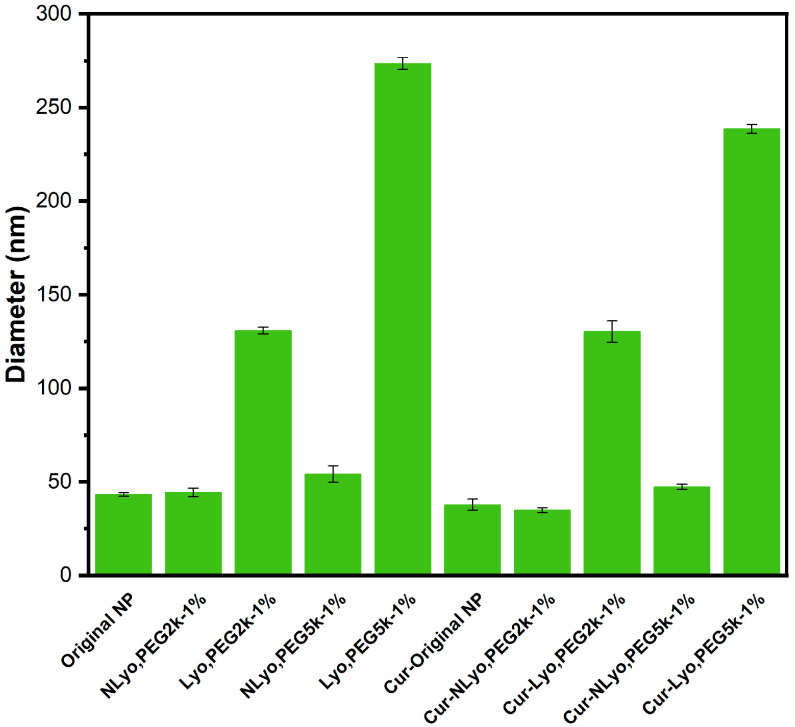
Systematic evaluation of PEG_2k_ and PEG_5k_ at 1% *w*/*v*: Hydrodynamic diameter of blank and curcumin-loaded nanoparticles was explored in the presence of PEG_2k_ and PEG_5k_ at 1% *w*/*v* and reported as Original (no lyophilization), NLyo (PEG cryoprotectant added but no lyophilization), and Lyo (cryoprotectant added and lyophilized).

Based on these results, PEG_2k_ and PEG_5k_ at 1% *w*/*v* concentration were further evaluated as cryoprotectants for blank and curcumin-loaded nanoparticles with or without lyophilization. The curcumin-loaded nanoparticles, Cur-Original NP, Cur-NLyo NP, and Cur-Lyo NP, are defined as Original (no cryoprotectant added/non-lyophilized), NLyo (cryoprotectant added/non-lyophilized), and Lyo (cryoprotectant added/lyophilized), respectively. Addition of cryoprotectants (1%, PEG_2k_ or PEG_5k_) to nanoparticle solutions without further lyophilization and redispersion has no effect on their size distributions. It was found to be true for both blank and curcumin-loaded formulations, suggesting no aggregation resulting from cryoprotectant addition. Upon lyophilization and redispersion, the sizes of nanoparticles containing PEG_5k_ for both blank and curcumin-loaded samples were larger than 200 nm, while the ones lyophilized and redispersed with PEG_2k_ (blank and curcumin-loaded) were less than 200 nm, suitable for biomedical applications (Figure 2 and Table S2).

**Figure 3:**
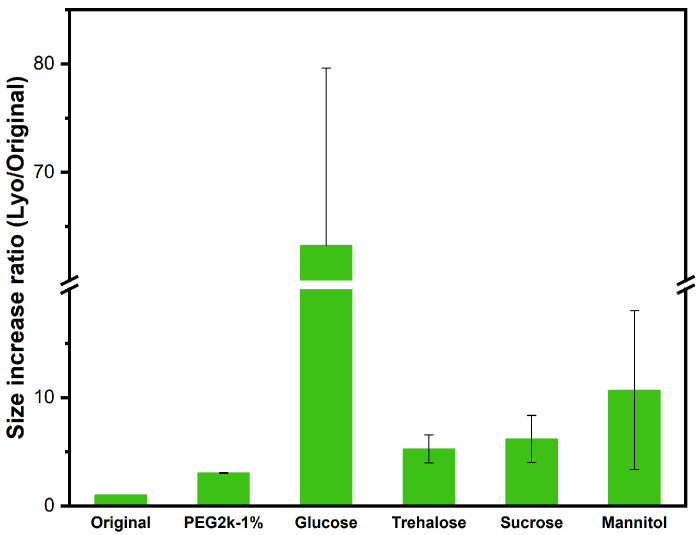
The size increase ratio of blank lyophilized nanoparticles containing 1% *w*/*v* of PEG_2k_, glucose, trehalose, sucrose, and mannitol as cryoprotectant relative to original nanoparticles. The size of Lyo nanoparticles was normalized to the size of original nanoparticles (first bar, non-lyophilized original nanoparticles) to evaluate the efficacy of each cryoprotectant on physical stability. All measurements were carried out in triplicate.

Considering that 1% *w*/*v* was the optimal concentration of cryoprotectant for nanoparticles, we also evaluated alternative cryoprotectants: trehalose, glucose, mannitol, and sucrose at 1% *w*/*v* concentration. It should be noted that higher concentrations of sugars were not tested in this study, as the major goal here was the comparative analysis of different sugars with PEG at its optimal concentration. There are studies devoted to sugars that have also shown that depending on the lyophilizate, lower concentrations of sugars may in fact be more suitable in retaining the stability of nanoparticles [46,51,55]. The relevant data are summarized in Figure 3 and Table S3. Nanoparticles containing sugars exhibited larger relative sizes compared to PEG_2k_, and there was higher variability from one batch to another. Our results agree well with those reported earlier. For example, Le et al. studied the lyophilization of polyelectrolyte nanogels using sugars at 1% *w*/*v* concentration. They reported that all sugar-based cryoprotectants increased the nanoparticle size upon lyophilization and redispersion; trehalose was the most and glucose the least effective cryoprotectant [46].

**Figure 4:**
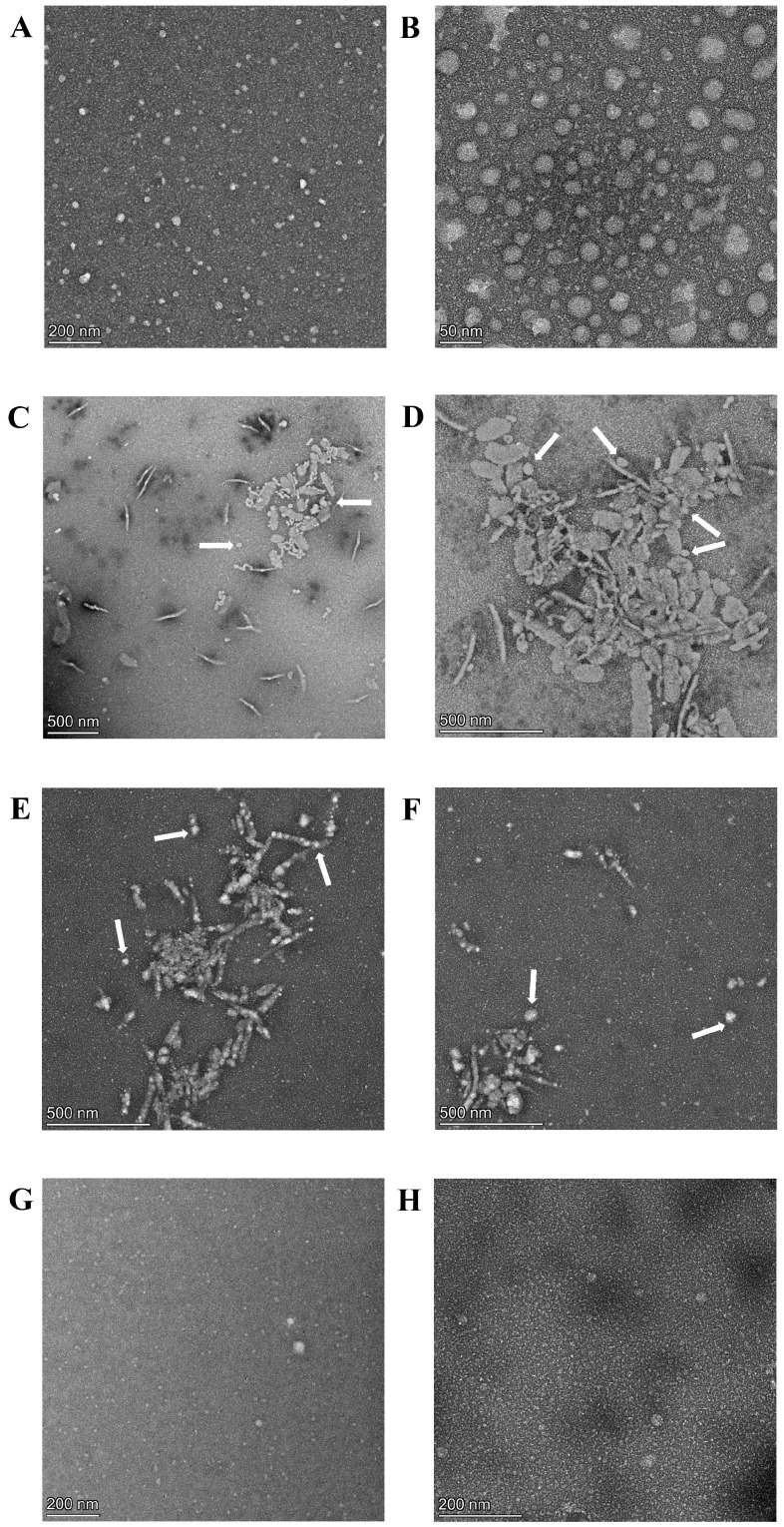
TEM images of nanoparticles: (**A**,**B**) represent Original blank nanoparticles with different magnifications, depicting spherical morphology; (**C**,**D**) are for lyophilized and redispersed blank nanoparticles containing glucose 1% *w*/*v* as cryoprotectant with different magnifications; (**E**,**F**) are for lyophilized and redispersed blank nanoparticles with trehalose 1% *w*/*v* as cryoprotectant. White arrows show spherical nanoparticles are surrounded by cryoprotectant-induced rod-type aggregates; (**G**,**H**) represent lyophilized and redispersed blank and curcumin-loaded nanoparticles, respectively, with PEG_2k_ at 1% *w*/*v* as cryoprotectant.

Nanoparticles containing cryoprotectants including PEG_2k_, glucose, and trehalose were evaluated further using TEM. Based on our results (Figure 4), we determined the following: (i) the original blank nanoparticles are spherical in nature with uniform size distribution (Figure 4A,B with Figure 4B as a magnified image of Figure 4A); (ii) there was aggregation in lyophilized and redispersed nanoparticles containing glucose (Figure 4C,D with Figure 4D as a magnified image of Figure 4C) and trehalose (Figure 4E,F); and (iii) there was no significant difference between original and lyophilized/redispersed nanoparticles containing PEG_2k_, with no sign of aggregation (Figure 4G representing blank nanoparticles and Figure 4H showing curcumin-loaded nanoparticles). The observed aggregation in nanoparticles containing glucose and trehalose is in agreement with the size variations and larger sizes seen during DLS analyses. Rod-type aggregation around spherical nanoparticles was visible in these images, as shown by the arrows in Figure 4C–F. TEM analysis together with DLS measurements further validated PEG_2k_ as an optimal cryoprotectant for nanoparticles. Our findings are consistent with a previously reported study about the evaluation of different cryoprotectant for PEG-PLA-based polymeric micelles. PEG was shown to be more efficient than saccharides in enhancing the integrity of micelles after reconstitution for copolymers containing a higher ratio of PLA/PEG content. For amphotericin-loaded PEG_5k_-PLA_15k_ micelles, the optimum concentration of PEG_2k_, PEG_4k_, and PEG_6k_ were 20, 20, and 10 mg/mL, respectively [56].

**Figure 5:**
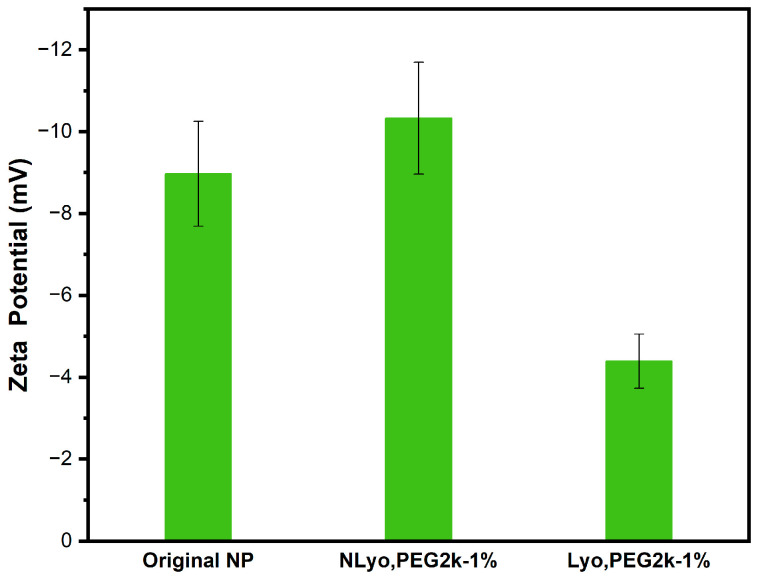
Zeta potential analysis of blank nanoparticles containing PEG_2k_ at 1% *w*/*v* in triplicate: the effect of cryoprotectant addition and lyophilization process on the surface charge of nanoparticles were assessed at different stages including Original, NLyo, and Lyo nanoparticles.

In order to study the effects of cryoprotectant addition on the surface charge of nanoparticles, we evaluated Original, NLyo, and Lyo nanoparticles containing 1% *w*/*v* PEG_2k_ (Figure 5). The zeta potential analysis demonstrated that the Original nanoparticles and NLyo nanoparticles had nearly identical surface charges. In contrast, the Lyo nanoparticles, upon redispersion, showed lower surface charge, likely resulting from a change in the polymeric arrangements on the surface of nanoparticles due to increased interaction between cryo-PEG chains and the nanoparticles, which might lead to the formation of a pseudo shell on the nanoparticles.

Zeta potential modulates protein adsorption and cell entry. Patil et al. have reported that a positive charge boosts bovine serum albumin adsorption, whereas a negative charge favors uptake by lung epithelial cells [57]. Ross et al. noted an optimum Caco-2 permeability and viability at a zeta potential near −12 millivolts in media [58]. Walkey et al. provide further details as to how the surface charge, augmented by polyethylene glycol density or exposed amines, interacts with protein corona composition to affect cell association [59]. We believe that in situ characterization, using methods such as dynamic light scattering and electron microscopy in physiologically relevant media, combined with the systematic evaluation of the size, surface charge, and chemistry, could address more clearly the link between these nanoparticle properties and biological outcomes.

### 2.3. Effect of Cryoprotectant on the Drug Release Profiles

**Figure 6:**
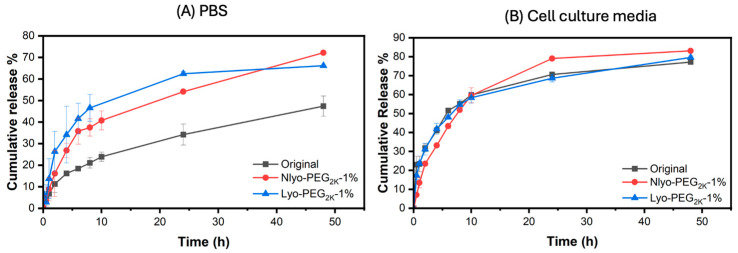
Evaluation of the effect of PEG_2k_-1% addition and lyophilization on curcumin release for Original, NLyo, and Lyo nanoparticles over 48 h: (**A**,**B**) illustrate the drug release profile conducted in PBS buffer and cell culture media, respectively. Drug release studies were carried out in PBS buffer and cell culture media containing 1% *v*/*v* Tween 80 using dialysis, and all measurements were performed in triplicate.

Curcumin, a naturally occurring polyphenol with varied medicinal efficacy [60], was used, as a hydrophobic drug model, to assess the impact of cryoprotectant addition and lyophilization on the drug release profile of nanoparticles. The curcumin encapsulation efficiency and curcumin loading capacity of nanoparticles were found to be 74% and 3.57%, respectively. PEG_2k_ at 1% *w*/*v* was used as a cryoprotectant to evaluate the drug release profile in PBS and cell culture media. The release studies conducted in PBS buffer revealed that the presence of 1% cryo-PEG in both non-lyophilized (NLyo) and lyophilized and then redispersed (Lyo) samples, enhances curcumin release from the nanoparticles. For example, over 24 h, only 34% of Cur is released from the Original nanoformulation (Figure 6A, black curve), while in the NLyo or Lyo samples containing 1% PEG_2k_, it is 54% (Figure 6A, red curve) and 62% (Figure 6A, blue curve), respectively. This indicates an enhancement of ~1.6 and 1.8 times with respect to the Original nanoparticles. A similar increase is noted over a 48 h period. This could be due to the ability of cryo-PEG chains, with their hydrophobic pockets, to stabilize curcumin and alter its distribution within nanoparticles, primarily shifting it towards the shell, which makes curcumin more readily available for release. However, the release study carried out in cell culture media with serum exhibited (i) a slightly faster release in Original nanoparticles compared to the one in PBS buffer (Figure 6A,B, blue curves) and (ii) nearly similar drug release for all three samples, regardless of cryoprotectant addition or lyophilization process (Figure 6B, Original, NLyo and Lyo). All three measured in cell culture media had about 75% cumulative release after 48 h, which was similar to the cumulative release in the cryo-PEG added samples (NLyo and Lyo) studied in PBS buffer. The interaction of proteins present in the cell culture media with nanoparticles may be dominated, thereby mitigating the interactions of nanoparticles with the added cryo-PEG chains. These interactions seem to increase curcumin release in all the samples (Figure 6B). Overall, at a concentration of 1% *w*/*v*, PEG_2k_ seems to have no impact on the curcumin release behavior in cell culture media.

### 2.4. Drug Release Kinetics Study

We subsequently evaluated the release kinetics of curcumin from Original, NLyo, and Lyo nanoformulations. For release studies in PBS (Table 1), the Baker–Lonsdale model provided the best fit for the Original nanoformulation with the highest R^2^ value of 0.9981. The Korsmeyer–Peppas and Weibull followed closely, with R^2^ values of 0.9978 and 0.9975, respectively, requiring the AIC values to be compared for finding the best fit. The Baker–Lonsdale model revealed the lowest AIC value of 14.20 confirming the best fit for Original nanoparticles. The Weibull model with the highest R^2^ was found to be the best fit for NLyo and Lyo nanoparticles. Our data suggest that the drug release from Original nanoparticles, which follows the Baker–Lonsdale model, is mainly controlled by diffusion. For NLyo and Lyo nanoparticles, the drug release that followed the Weibull model, could be attributed to a combination of release mechanisms including diffusion and relaxation. For a better understanding of the release mechanism in Weibull model, the β values for nanoparticles were evaluated. The β values were found to be 0.538, 0.536, and 0.352 for the Original, NLyo-1%PEG_2k_, and Lyo-PEG_2k_ 1%, respectively. Since all values were below 0.75, these suggest that the release behavior can be mainly attributed to Fickian diffusion, where transport is primarily governed by a concentration gradient. The most important parameters by which the suitable model was selected are highlighted in the red boxes in Table 1.

In cell culture media (Table 2), the Weibull model again showed the best fit across all formulations, with R^2^ values ranging from 0.9166 to 0.9542. The β values (0.441, 0.544, and 0.349) stayed within the same range, reinforcing that the release was still diffusion controlled. Overall, our results suggest that diffusion is the primary mechanism of drug release in both PBS and cell culture media and indicate that presence of the cryoprotectant and lyophilization process do not significantly alter the release kinetics parameters. The highest R^2^_adj values for all three nanoformulations confirming the Weibull model are highlighted in red boxes in Table 2.

### 2.5. Biological Evaluation of Cryoprotectant Addition and Freeze-Drying Effects

We previously reported that exposure to free curcumin can be cytotoxic to endothelial cells [61]. To further investigate this effect and compare the potential benefits of nanoparticle encapsulation across different formulations, the viability of HUVEC (human umbilical vein endothelial) cells was assessed following a 24 h exposure to free curcumin, curcumin-loaded nanoparticles (at equivalent curcumin concentrations), and blank nanoparticles. Cell viability was measured using the MTT assay, which evaluates the mitochondrial metabolic activity via a colorimetric readout. All measurements were normalized to untreated control cells.

**Figure 7:**
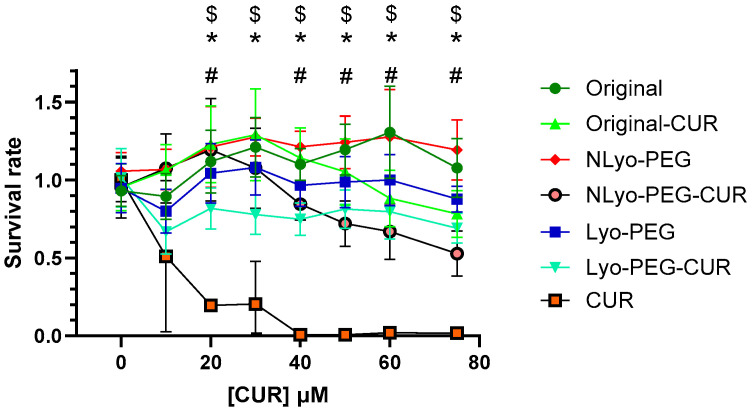
Cell viability of HUVEC endothelial cells assessed by MTT assay after 24 h of exposure to free curcumin (Cur), Cur-loaded nanoparticles (Original-Cur, NLyo-PEG-Cur, and Lyo-PEG-Cur), or blank nanoparticles (Original, NLyo-PEG, and Lyo-PEG). Data are expressed as mean ± SD from n = 5 biological replicates and were analyzed using two-way ANOVA. $ *p* < 0.05 for Original-CUR vs. Cur. * *p* < 0.05 for NLyo-PEG-Cur vs. Cur. # *p* < 0.05 for Lyo-PEG-Cur vs. Cur. n = 5.

As shown in Figure 7, exposure to free curcumin at concentrations equal to or exceeding 20 μM led to a significant decrease in HUVEC cell viability and complete loss of detectable viable cells at 40 μM and above. In contrast, exposure to all curcumin-loaded nanoparticle formulations at concentrations of 20 μM or higher resulted in improved HUVEC cell viability compared to treatment with free curcumin at the equivalent concentration, thus indicating a protective effect of the nanoparticle delivery systems.

Moreover, exposure to all nanoparticle formulations did not induce statistically significant cytotoxicity compared to the untreated control cells, with the sole exception of NLyo-PEG-Cur at 75 μM. No statistical difference in cell viability was observed between NLyo-PEG-CUR and Lyo-PEG-CUR, indicating that the lyophilization process did not introduce any toxic effects. Additionally, comparisons between Original vs. NLyo-PEG and Original-CUR vs. NLyo-PEG-CUR formulations revealed no statistically significant differences, indicating that addition of PEG as a cryoprotectant did not contribute to toxicity. These findings are consistent with a study assessing the toxicity of solid lipid nanoparticles supplemented with 5% mannitol, which reported no cryoprotectant-induced toxicity [62].

**Figure 8:**
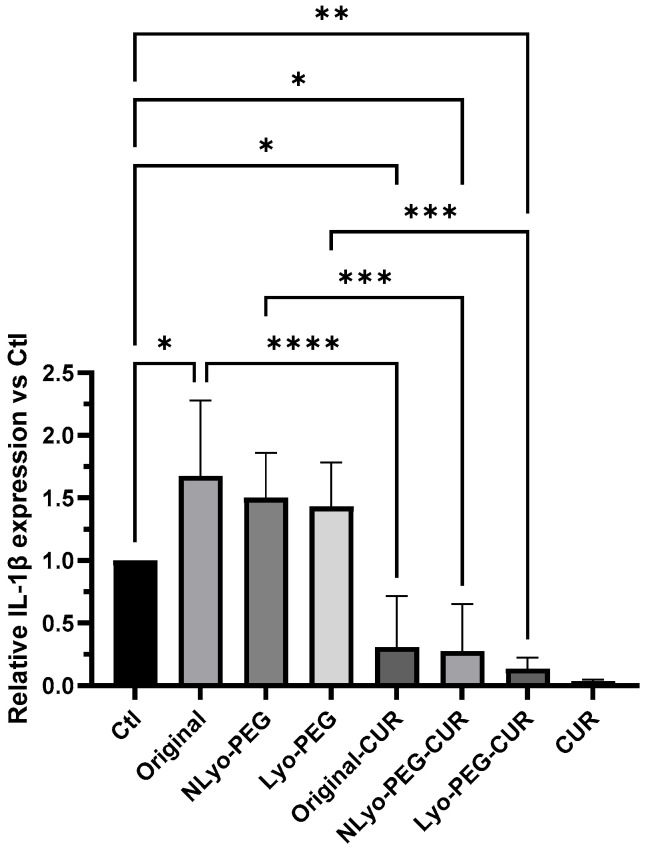
Anti-inflammatory activity of curcumin (Cur) assessed by quantification of interleukin-1β (IL-1β) secretion in THP-1 macrophages. Cells were pretreated for 24 h with 50 μM free Cur, Cur-loaded nanoparticles, or blank nanoparticles followed by stimulation with 10 μM nigericin for 30 min. IL-1β levels were analyzed by Western blotting. “Ctl” refers to cells treated with nigericin alone. Data represent n = 3 independent experiments and were analyzed using one-way ANOVA. * *p* < 0.05, ** *p* < 0.01, *** *p* < 0.001, **** *p* < 0.0001.

Curcumin concentrations ranging from 2 to 50 µM have been widely used in macrophage cultures in vitro, consistently demonstrating anti-inflammatory and antioxidant effects without significant cytotoxicity for this cell type. We and others have previously demonstrated that this concentration range effectively modulates key inflammatory mediators, including TNF-α, IL-6, and IL-1β, across various cellular models, such as THP-1-derived macrophages [63,64]. To evaluate the anti-inflammatory activity of curcumin and curcumin-loaded nanoparticles, the inflammasome pathway was activated in THP-1 macrophages using nigericin, a well-established inflammasome activator that induces the secretion of the pro-inflammatory cytokine, interleukin-1β (IL-1β). Figure 8 shows that pretreatment of the cells with NLyo-PEG and Lyo-PEG nanoparticles had no significant effect on IL-1β secretion, while pretreatment with 50 μM free Cur, Original-Cur, NLyo-PEG-Cur, or Lyo-PEG-Cur significantly decreased IL-1β secretion by 96%, 69%, 72%, and 86%, respectively. The unmodified original nanoparticles induced a statistically significant increase in IL-1β secretion; however, this increase was no longer significant in nanoparticle formulations containing PEG. There was no statistical difference in IL-1β secretion level between free Cur and the Cur-loaded nanoparticles, regardless of lyophilization, indicating that the cryoprotectant and freeze-drying process did not impair the anti-inflammatory activity of the encapsulated curcumin. A similar trend was observed in a study investigating the influence of trehalose on the antimicrobial property of gallium protoporphyrin-loaded lipid nanoparticles, where the cryoprotectant did not compromise the functional efficacy of the formulation [65].

## 3. Materials and Methods

### 3.1. Blank and Curcumin-Loaded Nanoparticle Preparation

The co-solvent evaporation method was utilized for the preparation of blank and curcumin-loaded nanoparticles. Briefly, for the preparation of blank nanoparticles, 5 mg of AB_2_ polymer was dissolved in 2 mL of HPLC grade acetone. The organic phase was added dropwise into 2 mL of Milli-Q water at the rate of 1 drop every three seconds while being stirred at 400 RPM overnight. Upon complete evaporation of acetone, the nanoparticles were filtered using a PVDF syringe filter with a 0.22 μm pore size to remove any aggregated particles.

Curcumin-loaded nanoparticles were prepared following the same procedure with the additional step of co-dissolving 0.5 mL of curcumin stock solution (1 mg/mL) in acetone along with AB_2_ polymer in a 1:10 ratio, followed by degassing.

### 3.2. Dynamic Light Scattering (DLS) and Zeta Potential Analyzer

The Brookhaven instrument (NanoBrook Omni, Holtsville, NY, USA) was employed to assess the nanoparticles’ hydrodynamic diameter, size distribution, and zetapotential before and after adding cryoprotectants, as well as the followed lyophilization and redispersion process. The equipment features a 40 mW diode laser (640 nm) and operates at a detection angle of 90°. All measurements were performed in triplicate, with each measurement comprising 4 cycles, each lasting 2 min.

### 3.3. Transmission Electron Microscopy (TEM)

The overall physical characteristics of nanoparticles (before and after lyophilization in the presence of cryoprotectants) including their morphology, size, size distribution, the potential aggregation induced by cryoprotectant addition, or incomplete redispersion of nanoparticles were evaluated using TS Talos F200X TEM (Thermo Fisher Scientific, Waltham, MA, USA). To prepare TEM grids, carbon-coated copper TEM grids were glow discharged at 10 mA for 10 s. Then, 5 μL of 5 times-diluted nanoparticles was applied to the carbon side of the TEM grid; after 5 min, the excess water was removed using a paper. To enhance the contrast of TEM images, 5 μL of 2% uranyl acetate solution was added to the grid, and the excess was removed after 45 s. Grids were air-dried prior to imaging.

### 3.4. Freeze-Drying Procedure

A FreeZone 2.5 Liter-50C (Labconco, Kansas City, MO, USA) benchtop freeze dryer was used to carry out the freeze-drying process. In a standard experiment, a desired concentration of selected cryoprotectant was added to 2 mL of nanoparticles with a concentration of 2.5 mg/mL. The mixture was vortexed gently for 2 h at room temperature to ensure the uniform distribution of cryoprotectant among nanoparticles. Nanoparticles were then frozen at −30 °C overnight, followed by a 24 h drying process in the lyophilizer. The lyophilizate was reconstituted to the original concentration of nanoparticles by adding 2 mL of milli-Q water dropwise. As the final step, redispersed nanoparticles were vortexed at a mild speed at room temperature for 2 h prior to analysis.

### 3.5. Drug Loading and Release Studies

The encapsulation efficiency (EE) and drug loading percentage (DL) of nanoparticles were obtained using following equations, referring to the curcumin calibration equation obtained by plotting the absorbance of curcumin at 425 nm against different curcumin concentrations in the range of 0.98–31.25 μg/mL in methanol. In a typical measurement, 30 μL of curcumin-loaded nanoparticles was diluted 40 times with methanol, and the corresponding absorbance was then measured.

$$EE\%=\frac{Encapsulated\;Drug}{Total\;Drug\;used}\times 100$$

$$DL\%=\frac{Encapsulated\;Drug}{Polymer+Drug}\times 100$$

The dialysis method was used to study the curcumin release profile and the potential impacts of cryoprotectant addition and lyophilization process on the drug release kinetics. Briefly, 2 mL of nanoparticle suspension was placed into a Spectra/Por 3 (Spectrum Chemical, Waltham, MA, USA) dialysis membrane (Standard RC, 3.5 kDa MWCO) and dialyzed against 140 mL of phosphate-buffered saline (PBS, pH = 7.4, 0.01M, 37 °C) supplemented with 1% *v*/*v* Tween 80. Then, 40 μL of nanoparticles was aliquoted from the inside of the dialysis tubing at specified time intervals over a 48 h period, and the corresponding unreleased curcumin was measured by the 40 times dilution of nanoparticles with methanol. The cumulative release percentages were then calculated using the following equation:

$$Cumulative\;Release\;\%=\left(1-\frac{Unreleased\;Drug}{Initial\;Encapsulated\;Drug}\right)$$

In order to evaluate the influence of the cryoprotectant and lyophilization on the release profile of nanoparticles, three sets of nanoparticles were prepared for comparison: (i) Original nanoparticle; (ii) nanoparticle + 1% *w*/*v* of PEG_2k_ (non-lyophilized; NLyo); (iii) nanoparticle + 1% *w*/*v* of PEG_2k_ (Lyophilized; Lyo). Curcumin release was studied against PBS buffer at 37 °C.

To further investigate the impact of the cryoprotectants and lyophilization on the release profile in a more biologically relevant context, the cell culture media (RPMI 1440, 1×), supplemented with 10% FBS and 1% *v*/*v* Tween 80, was used as release media instead of PBS at 37 °C. Moreover, the three aforementioned sets of nanoparticle formulations were mixed with cell culture media with a 3:1 *v*/*v* ratio and vortexed gently for 10 min prior to the release study, simulating curcumin release in the cell culture media. At designated time intervals over 48 h, 40 μL of nanoparticles was collected from the inside of the dialysis tubing. Nanoparticles were diluted 40 times with methanol and incubated for 6 h at room temperature before UV–Vis measurements. To separate the precipitated cell media proteins upon methanol addition, the diluted samples were centrifuged at 10,000 RPM for 10 min, and the supernatant solution was used for the measurement.

### 3.6. Drug Release Kinetics Study

Release kinetics was evaluated to unravel the drug release mechanism of nanoparticle formulations with and without cryoprotectants in PBS and cell culture media. DDSolver was used to conduct release kinetics evaluations. Seven models in DDSolver including Zero-order, First-order, Higuchi, Hixson–Crowell, Korsmeyer–Peppas, Baker–Lonsdale, and Weibull were tested. Each model was applied to the release data studied in PBS and cell culture medium. The fit was assessed using R^2^, and for the Korsmeyer–Peppas and Weibull, the n and β values were applied to interpret the release mechanism, as well [66,67].

### 3.7. Cell Viability Assay

Human umbilical vein endothelial cells (HUVEC, ATCC #CRL-1730) were used between passages 5 and 8. Cells were cultured in EGM-2 medium (Lonza Cat. No. CC-3162), seeded at a density of 5000 cells per well in 96-well plates, and incubated at 37 °C in a humidified atmosphere containing 5% CO_2_. After 24 h, cell culture media were replaced with fresh media containing nanoformulations with curcumin concentrations ranging from 0 to 80 μM, and cells were incubated for an additional 24 h. Cell proliferation was assessed using the MTT assay, which measures the mitochondrial activity in viable cells. Following treatment, the cells were washed with PBS and incubated with 100 µL of a 0.5 mg/mL solution of 3-(4,5-dimethythiazol-2-yl)-2,5-diphenyltetrazolium bromide prepared in EGM-2 for 4 h in the dark. After incubation, the media were replaced with 100 μL DMSO to solubilize the formazan crystals. The absorbance was measured at 575 nm using a Synergy H1 plate reader (BioTek, Winooski, VT, USA), and the survival rate was obtained by normalization with measured optical densities from untreated cells.

### 3.8. Quantification of Interleukin-1b Secretion by Western Blotting

Human monocytic THP-1 cells (ATCC #TIB-202) were used between passages 6 and 10 and cultured in RPMI 1640 medium (Wisent, Cat. No. 350-000-CL, Saint-Jean-Baptiste de Rouville, QC, Canada), supplemented with 10% fetal bovine serum (FBS, Wisent, Cat. No. 090150), at 37 °C in a humidified atmosphere containing 5% CO_2_. Cells were seeded at 750,000 cells/mL (1 mL per well) in 12-well plates and treated with phorbol 12-myristate 13-acetate (PMA, Millipore Sigma, Cat. No. P1585, Burlington, MA, USA) at 100 ng/mL to induce their differentiation into macrophages. After 72 h, the medium was replaced, and the adhered macrophages were pretreated with Cur or an equivalent Cur concentration of nanoparticles of 50 μM or an equivalent volume of medium (control). After 24 h, the media were replaced with serum-free medium supplemented with nigericin at 10 μM for 30 min or an equivalent volume of medium (control nigericin). At the end of the treatment, media were collected for immunoblotting analyses. Immunoblotting was performed using 45 μL of the conditioned media, and the samples were electrophoresed using 12% TGX Stain-Free gels (Bio-Rad, Mississauga, ON, Canada). Separated proteins were then transferred to a PVDF membrane overnight at 10 V. The membrane was blocked with 5% nonfat dry milk in standard TBST buffer, then incubated with an anti-IL-1β rabbit monoclonal antibody (dilution 1/2000, Cell Signaling, #83186, Danvers, MA, USA) overnight at 4 °C, washed in TBST, and incubated with an HRP-conjugated goat anti-rabbit antibody (dilution 1/10,000, ThermoScientific, #32460, Waltham, MA, USA). Protein detection was achieved using Western Lighting Ultra chemiluminescence substrate (Perkin Elmer, NEL10501EA, Waltham, MA, USA) to reveal and quantify the interleukin-1β (IL-1β) signal using a Chemidoc MP Imaging System (Bio-Rad, Mississauga, ON, Canada). Stain-free gels allowed for the quantification of total protein in acrylamide gel for normalization. Finally, the results were expressed as ratios of nigericin-exposed cells without treatment.

## 4. Conclusions

Polymeric soft nanoparticles from amphiphilic asymmetric branched star polymers (also referred to as miktoarm polymers) have offered an advantageous platform to address key issues in drug delivery. The lyophilization and redispersion of such nanoparticles are essential for extending the shelf life and formulating therapeutic interventions at desired concentrations upon demand. There are limited studies on the effects of freeze drying and aqueous reconstitution of the resulting solid nanoparticles on their physicochemical properties and biological efficacy. We evaluated nanoparticles from well-studied branched macromolecule compositions, containing a combination of FDA approved, biocompatible, and biodegradable polymeric arms, and curcumin, a natural polyphenol with diverse beneficial medicinal traits. Through an analysis of varied cryoprotectants at different concentrations, we demonstrated that polyethylene glycol, which is akin to the composition in the star polymer precursor, with a molecular weight of 2000 Da and at a concentration of 1% *w*/*v*, is ideal for the [46] redispersion of the freeze-dried nanoparticles. There was no effect of the addition of 1% PEG to nanoformulations with/without lyophilization on the curcumin release profiles in cell culture medium; while in PBS buffer, the drug release was accelerated due to PEG addition. Nanoformulations, before and after lyophilization/redispersion, exerted no significant change in cell viability of endothelial cells at varied concentrations and, in fact, protected cells from the cytotoxicity of free curcumin at concentrations of 20 μM or higher. Curcumin retains its anti-inflammatory activity in the nanoformulations, and curcumin loading into nanoparticles, freeze drying, and reconstitution processes did not cause any change in its biological activity. Our studies demonstrate that with a careful choice of the cryoprotectant at optimal concentrations, one can transform aqueous formulations from miktoarm star polymers into stable solid form with retention of their key properties. The significance of polyethylene glycol in nanoformulation development and enhanced circulation times upon their administration has been widely established. Our results suggest additional advantages of utilizing PEG during freeze drying and subsequent reconstitution of powders as aqueous dispersions. It is a more effective and better choice as a cryoprotectant especially for polymeric nanoparticles containing PEG as an integral part of the core–shell structure. During lyophilization and redispersion of the resulting powder form, interactions of cryoprotectant PEG with the shell surface of the nanoparticles helps enhance their stability and avoid collapse of their morphological integrity under stress and upon slow development to the original colloidal form.

## Figures and Tables

**Table 1:** Drug release kinetics model evaluation of curcumin-loaded NPs in PBS using DDSolver.

Model	Parameter	Original	NLyo	Lyo
**Zero-order**	R^2^_adj	0.5628	0.4662	0.1055
	AIC	68.78	80.5272	76.9778
	MSC	0.4196	0.2279	−0.3717
	k_0_	1.196	1.882	1.833
**First-order**	R^2^_adj	0.7349	0.8315	0.6463
	k_1_	0.018	0.044	0.062
	AIC	63.7871	68.9974	68.6274
	MSC	0.9197	1.3812	0.5561
**Higuchi**	R^2^_adj	0.9925	0.9575	0.8198
	AIC	28.1145	55.2121	62.5602
	MSC	4.4870	2.7594	1.2302
	k_H_	7.102	11.356	11.968
**Hixson**–**Crowell**	R^2^_adj	0.6813	0.7420	0.5146
	AIC	65.6262	73.2591	71.4762
	MSC	0.7358	0.9547	0.2396
	k_HC_	0.005	0.012	0.020
**Korsmeyer**–**Peppas**	R^2^_adj	0.9978	0.9676	0.9132
	AIC	16.4943	53.3254	56.7789
	MSC	5.6490	2.9481	1.8726
	k_KP_	8.145	13.954	19.745
**Baker**–**Lonsdale**	R^2^_adj	0.9981	0.9785	0.9078
	AIC	14.2003	48.4298	56.5281
	MSC	5.8784	3.4377	1.9005
	k_BL_	0.001	0.003	0.004
**Weibull**	R^2^_adj	0.9975	0.9921	0.9914
	AIC	18.8299	39.8791	36.5458
	MSC	5.4155	4.2927	4.1207
	β	0.538	0.536	0.352

**Table 2:** Drug release kinetics model evaluation of curcumin-loaded NPs in cell culture media using DDSolver.

Model	Parameter	Original	NLyo	Lyo
**Zero-order**	R^2^_adj	0.0110	0.3807	−0.4453
	AIC	86.6090	83.7011	90.2645
	MSC	−0.4371	0.0844	−0.9773
	k_0_	1.845	1.217	1.287
**First-order**	R^2^_adj	0.6578	0.7679	0.4341
	AIC	75.9956	73.8856	80.8882
	MSC	0.6243	1.0660	−0.0397
	k_1_	0.058	0.031	0.056
**Higuchi**	R^2^_adj	0.7977	0.8718	0.6205
	AIC	70.7397	67.9503	76.8926
	MSC	1.1498	1.6595	0.3599
	k_H_	11.760	9.319	10.394
**Hixson**–**Crowell**	R^2^_adj	0.5215	0.6606	0.2780
	AIC	79.3490	77.6863	83.3233
	MSC	0.2889	0.6859	−0.2832
	k_HC_	0.019	0.008	0.016
**Korsmeyer**–**Peppas**	R^2^_adj	0.8786	0.8936	0.9339
	AIC	66.4524	66.9126	60.2363
	MSC	1.5786	1.7633	2.0255
	k_KP_	18.750	13.692	24.795
**Baker**–**Lonsdale**	R^2^_adj	0.8860	0.9162	0.8022
	AIC	65.0041	63.7047	70.3783
	MSC	1.7234	2.0841	1.0113
	k_BL_	0.004	0.002	0.003
**Weibull**	R^2^_adj	0.9326	0.9166	0.9542
	AIC	61.2426	65.1366	57.2354
	MSC	2.0995	1.9409	2.3256
	β	0.538	0.536	0.352

## Data Availability

Data for this work are contained within the article and Supplementary Materials section.

## Supplementary Materials

The following supporting information can be downloaded at https://www.mdpi.com/article/10.3390/ijms262010015/s1.
