# Supplementary material for: Unraveling Lyophilization and Redispersion Effects on Miktoarm Polymer-Based Nanoformulations
Source: Int J Mol Sci. 2025 Oct 15;26(20):10015. doi: 10.3390/ijms262010015 (PMC12563623; doi:10.3390/ijms262010015)
Supplement: Supplementary file 1 [file ijms-26-10015-s001.zip › ijms-3869983-supplementary.pdf]

Supporting Information

Materials:

3,5-dihydroxybenzyl alcohol (99%), propargyl bromide (80% in toluene), potassium carbonate (K_2CO_3, ≥99.0%), magnesium sulfate ($MgSO_4$), tin (II)2-ethyl hexanoate ($Sn(Oct)_2$, 92.5-100%), Methoxy polyethylene glycol monomethyl ether ($mPEG_{2k}$, 2050 g/mol), triethyl amine (≥99.5%), 4-(Dimethylamino)pyridine (DMAP, ≥99.0%), p-toluene sulfonyl chloride (PTSCL, ≥98%), hydrochloric acid (HCl, 37%), sodium azide (NaN3, ≥99.5%), copper (I) bromide (CuBr, 98%), *N*,*N*,*N*′,*N*″,*N*″-pentamethyl diethylenetriamine (PMDETA, 99%), ethylenediaminetetraacetic acid disodium salt dihydrate (EDTA), were purchased from Sigma-Aldrich and used as received. Spectra/ Por3 dialysis membrane (Standard RC, 3.5 kDa MWCO) was obtained from Thermo-Fisher Scientific). Copper bromide was purified using acetic acid and washed with anhydrous ethanol and ether, respectively. ε-caprolactone monomer was distilled over calcium hydride prior to use. PVDF syringe filters (13 mm, 0.22 µm, non-sterile, SyringeFilter.com).

Synthesis and Characterization of Miktoarm Polymer:

The total reaction scheme for AB2-based miktoarm polymer synthesis is illustrated in figure S1. In brief, mono-methoxy terminated PEG_{2k} was tosylated (P3), which resulted in ^{1}H-NMR peaks at 7.81, 7.36, and 2.46 ppm confirming successful functionalization of PEG (Fig S7). It was subsequently converted into azide (P4), which was verified by the absence of the tosyl peaks in the ^{1}H-NMR (Fig S8) and the presence of the azide (N=N) IR peak at 2100 cm^{-1} (Fig S10). Along with the synthesis of PEG-azide, the core containing PCL (P2) was synthesized by propargylating 3,5-dihydroxy benzyl alcohol, followed by ring opening polymerization at the primary alcohol site. The presence of the azide group at the end of the PEG chain in P4 allows for the attachment of PEG to the PCL-containing core (P2) via a copper (I)-catalyzed alkyne-azide cycloaddition reaction. The synthesis of the desired branched polymer P5 was confirmed by the disappearance of the azide peak in IR (Fig S10); HNMR (with degree of polymerization of 44 for PEG and 23 for PCL) (Fig S10); and the shift of retention time for P5 (compared to P2) towards higher molecular weights in gel permeation chromatography analysis (S12).

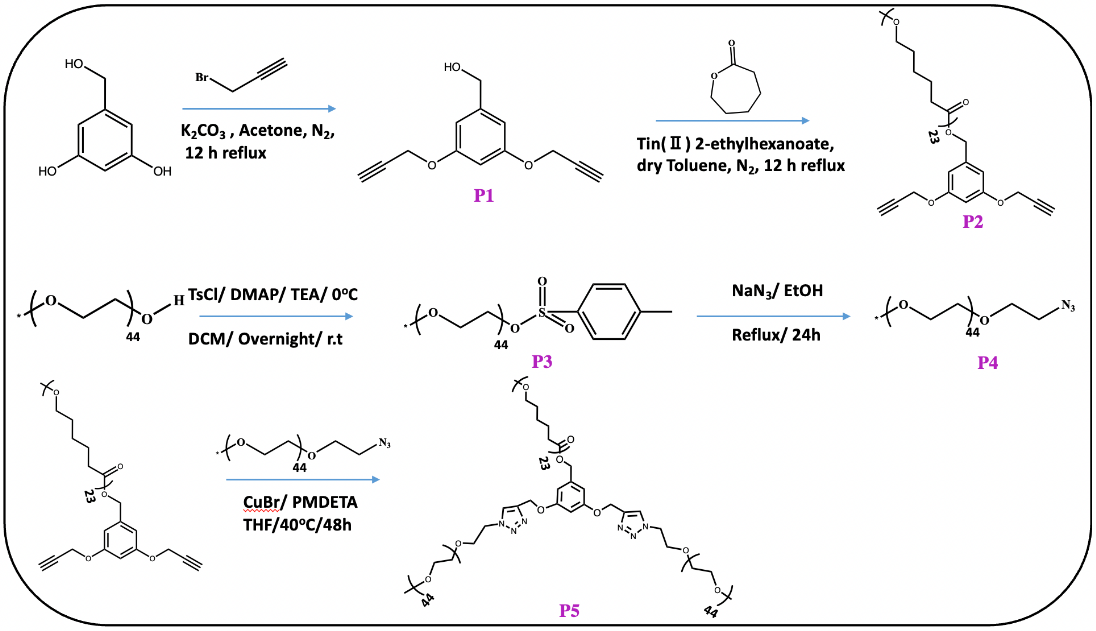

Figure S1 Reaction Scheme for AB2-based miktoarm polymer synthesis

Synthesis of (3,5-bis(prop-2-yn-1-yloxy) phenyl) methanol (P1): 3,5-dihydroxy benzyl alcohol (1.00 g, 7.14 mmol) and K_2CO_3 (1.28 g, 9.28 mmol) were dissolved in acetone, and then propargyl bromide (1.60 mL, 14.3 mmol) was added. The reaction was stirred overnight at 60°C under nitrogen atmosphere. After completion, the solvent was evaporated, and the reaction mixture was dissolved in dichloromethane (DCM) and washed three times with water. The DCM extract was dried over $MgSO_4$, and the solution was concentrated before being passed through the silica gel column with the eluent system of dichloromethane/ ethyl acetate: 20:1. The purified product was obtained as a yellow solid after the removal of solvent and being dried under the vacuum. Yield (0.60 g, 60%)

^{1}H NMR (500 MHz, $CDCl_3$): δ_H (ppm) 6.64 (2H, d, **a**), 6.56 (1H, t, **b**), 4.70 (4H, d, **c**), 4.68 (2H, d, **d**), 2.54 (2H, t, **e**), 1.70 (1H, t, **f**). ^{13}C NMR (500 MHz, $CDCl_3$): δ_C (ppm) 158 (**a**), 143 (**b**), 107 (**c**), 102 (**d**), 78 (**e**), 76 (**f**), 65 (**g**), 56 (**h**).

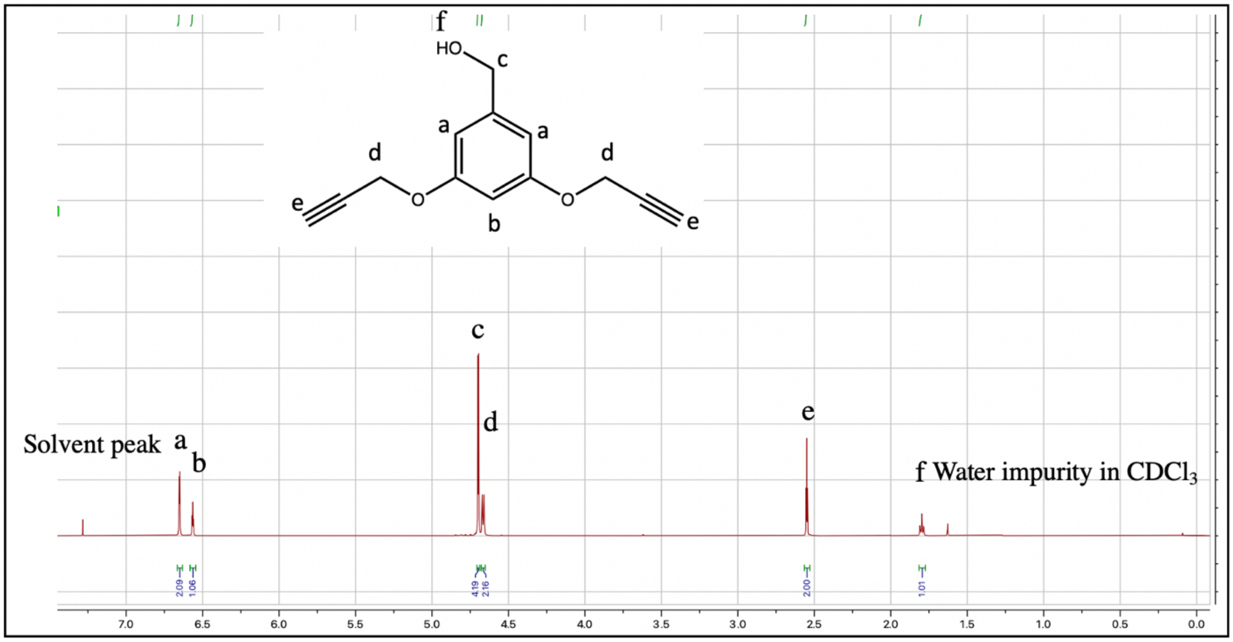

Figure S2 1-HNMR for P1

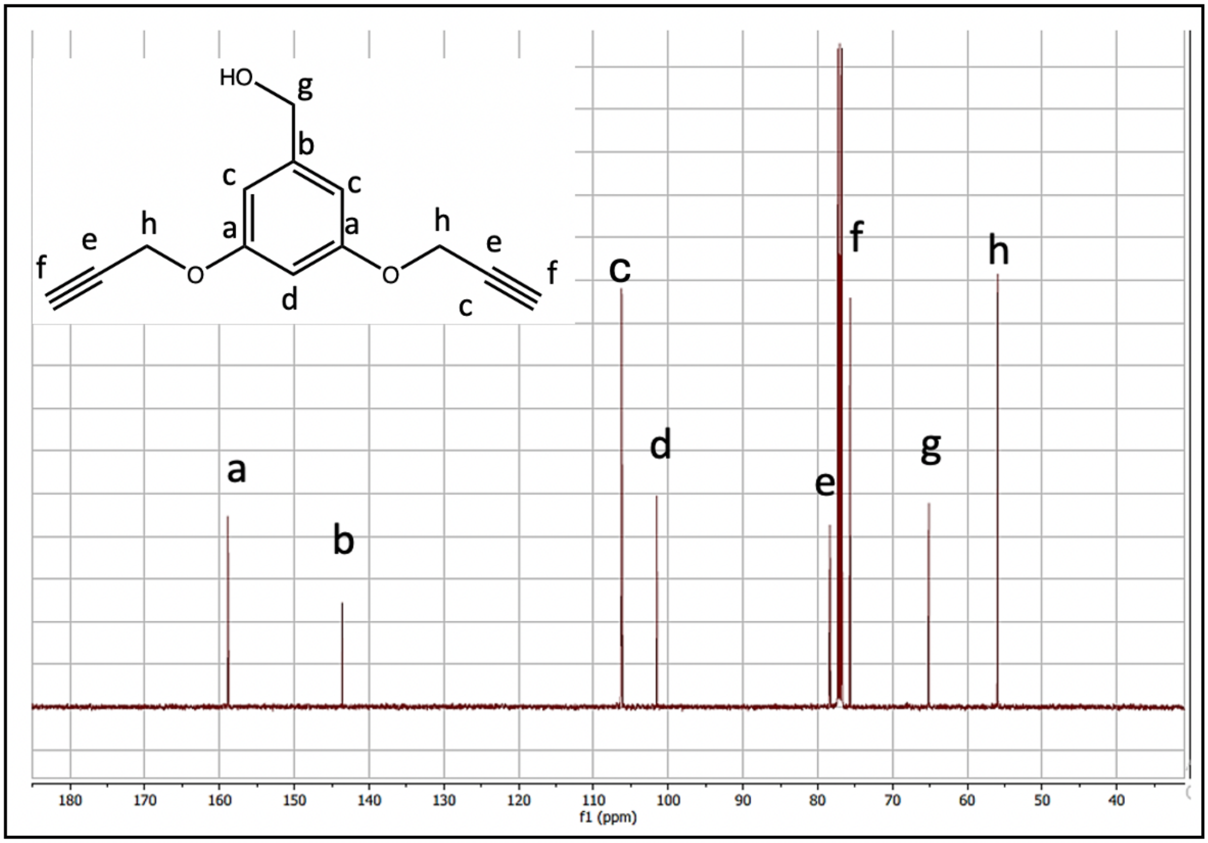

Figure S3 13CNMR for P1

Synthesis of (3,5-bis(prop-2-yn-1-yloxy) Benzyl PCL (P2):

Compound **1** (0.20 g, 0.93 mmol) and distilled caprolactone (2.07 mL, 18.7 mmol) were dissolved in dry toluene. The reaction mixture refluxed, and tin (II)2-ethyl hexanoate (0.08 mL, 0.19 mmol) was added at 90°C. The reaction mixture was further stirred at 105°C overnight. For the reaction workup, toluene was evaporated using rotary evaporator, and the crude product was dissolved in dichloromethane and precipitated into the cold diethyl ether. Product was obtained as a white powder and dried under high vacuum. (Yield: 1.30 g, 54%).

^1H NMR (500 MHz, $CDCl_3$): δ_H (ppm) 6.60 (2H, d, **a**), 6.57 (1H, t, **b**), 5.05 (2H, d, **c**), 4.69 (4H, d, **d**), 4.07 (45H, t, **e**), 3.66 (2H, t, e), 2.56 (2H, t, **f**), 2.40 (2H, t, **g**), 2.32 (45H, t, **g**), 1.66 (98H, m, **h, i**), 1.40 (48H, m, **k**). ^{13}C NMR (500 MHz, $CDCl_3$): δ_C (ppm) 174 (**a**), 158 (**b**), 138 (**c**), 107 (**d**), 103 (**e**), 77 (**f**), 76 (**g**), 66 (**h**), 65 (**i**), 63 (**j**), 56 (**k**), 34 (**l**), 28 (**m**), 25 (**n**), 24 (**o**). MS: MALDI-TOF (Mn= 2256 Da; Mw= 2696; PDI=1.08; degree of polymerization: 23)

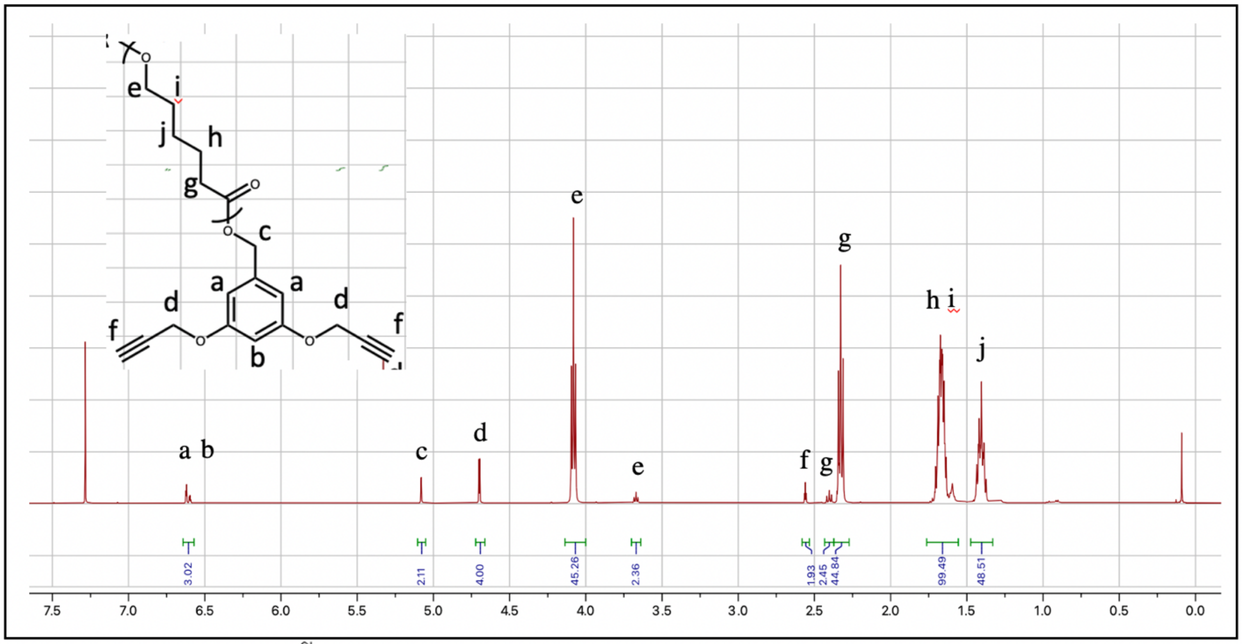

Figure S4 1HNMR for P2

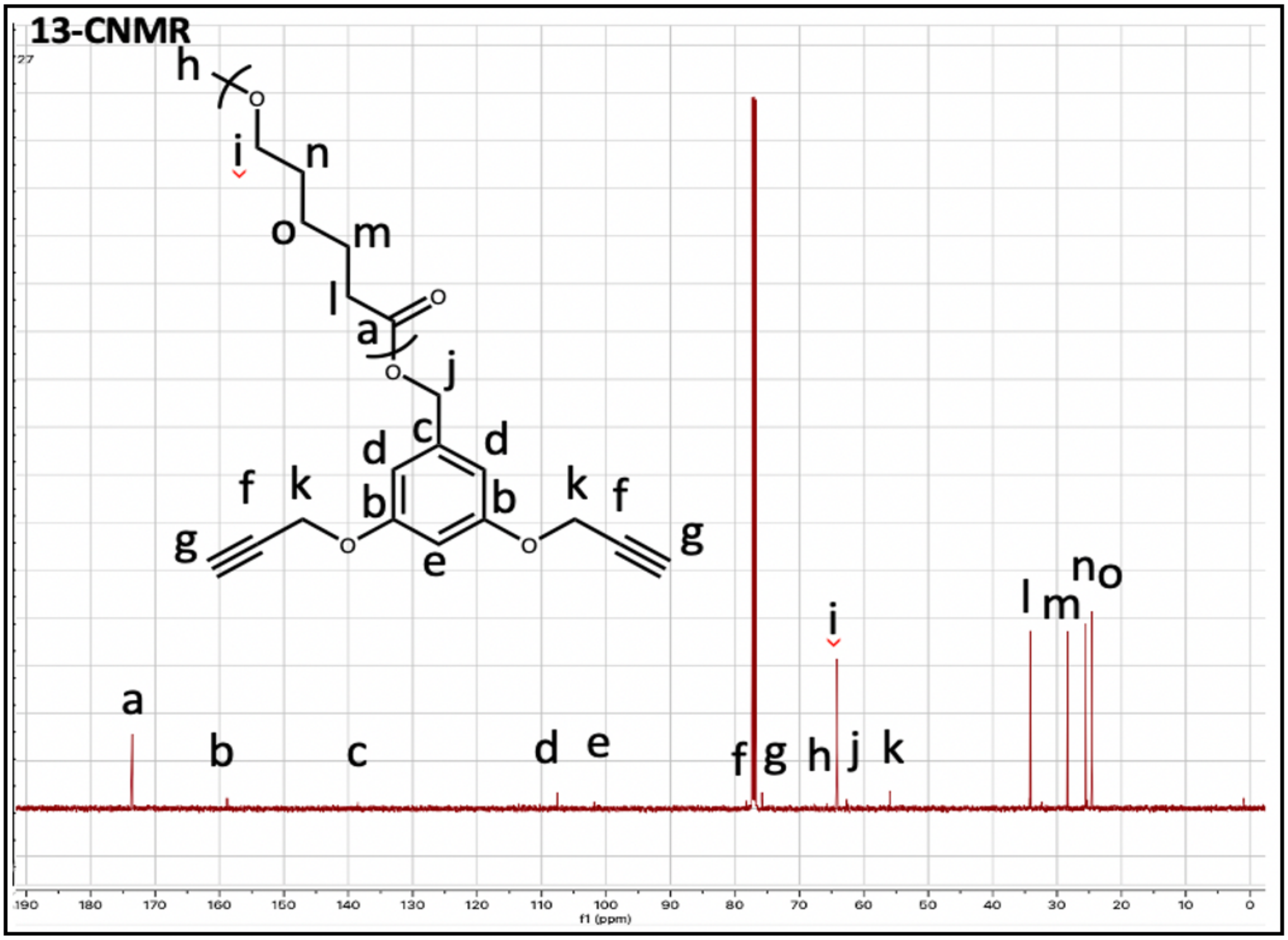

FigureS 5 13CNMR for P2

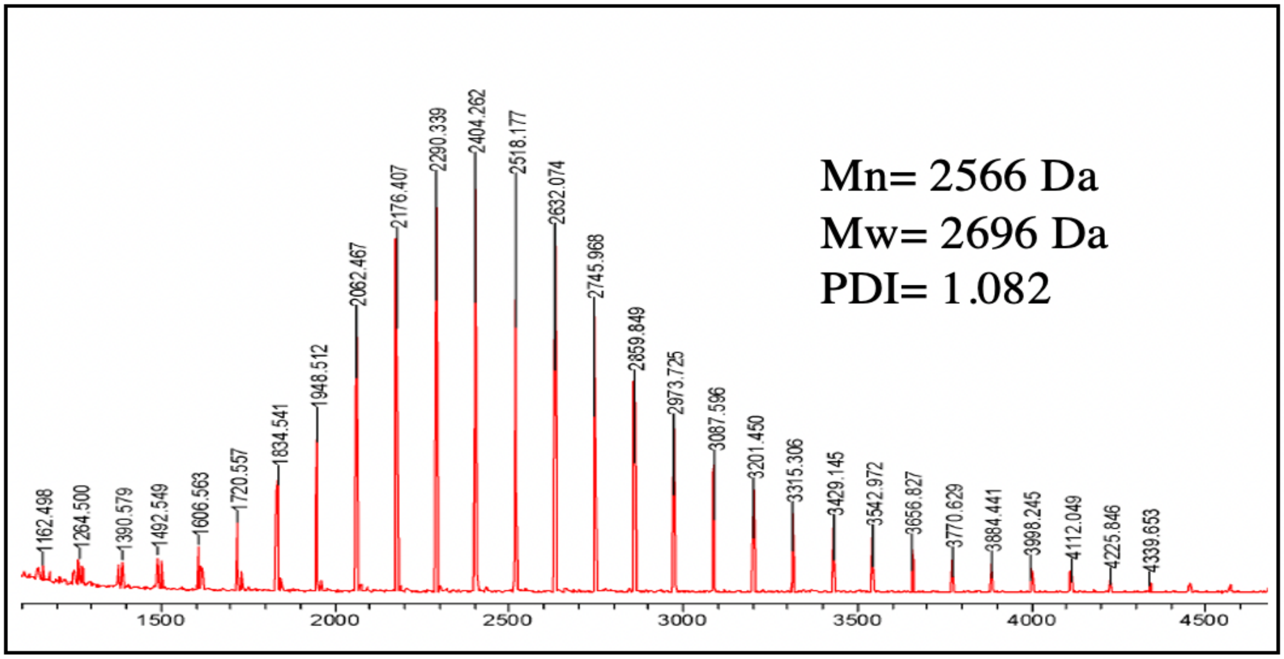

Figure S6 MALDI-TOF analysis of P2

Synthesis of mPEG$_{2k}$-OTs (P3):

mPEG$_{2k}$ (3.00 g, 1.50 mmol), triethyl amine (3.90 mL, 37.0 mmol), DMAP (46.0 mg, 0.377) were dissolved in dry dichloromethane and stirred for one hour at room temperature. The reaction flask was then placed into the ice bath, and pTSCL (1.20 g, 6.42 mmol) was added into the mixture. The reaction was further stirred overnight under nitrogen atmosphere at room temperature. For obtaining the product, reaction mixture was washed 4 times with HCl 0.1 M and dried over magnesium sulfate. The product solution in dichloromethane was precipitated into the cold ether, and the pure product was obtained as a white solid. (Yield: 2.10 g, 70%)

^{1}H NMR (500 MHz, $CDCl_3$): δ_H (ppm) 7.81 (2H, d, **a**), 7.36 (2H, d, **b**), 4.17 (2H, t, **c**), 3.65 (186H, t, **c**), 3.39 (3H, s, **d**), 2.46 (3H, s, **e**). Degree of polymerization: 46.

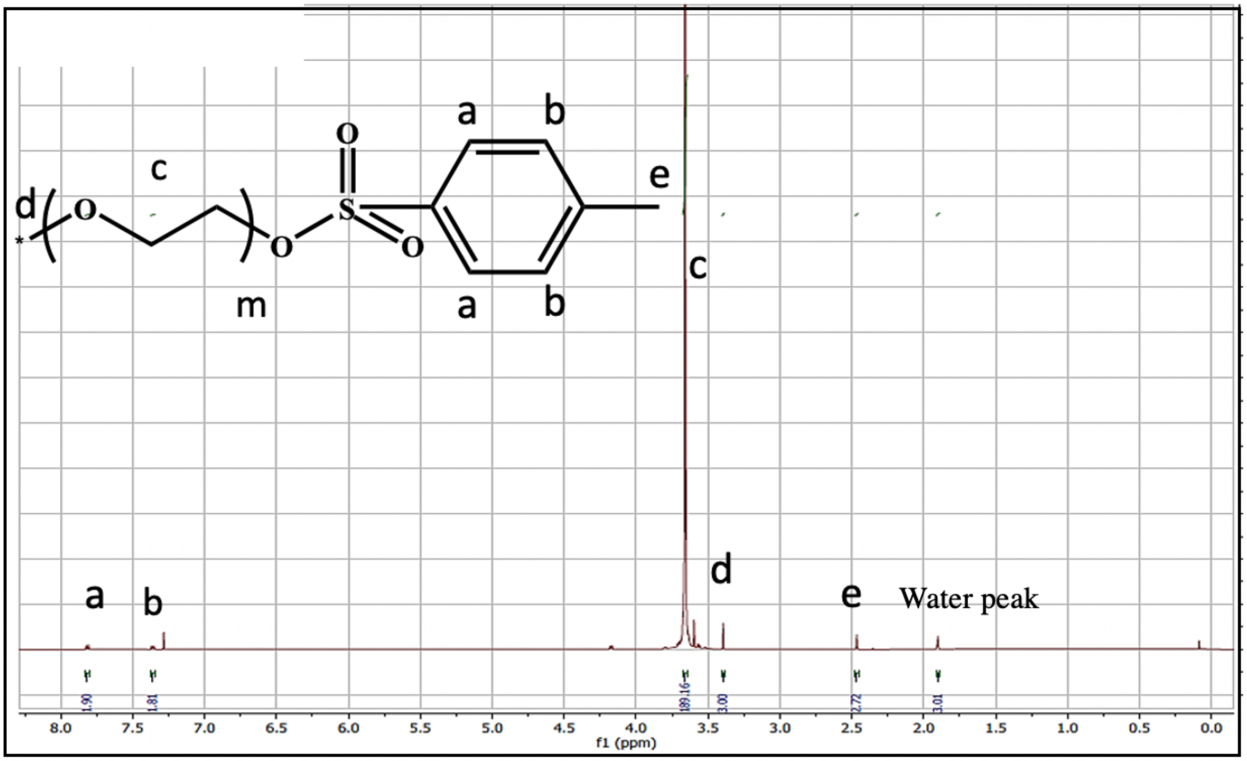

Figure S7 1HNMR for P3

Synthesis of PEG$_{2k}$-Azide (P4):

Compound **3** (2.00 g, 1.00 mmol) and sodium azide (214.5 mg, 3.3 mmol) were dissolved in anhydrous ethanol, The reaction was refluxed at 78°C for 24 hours under nitrogen atmosphere. Upon reaction completion, mixture was cooled down at room temperature, and then the flask was opened to air for half an hour. At the next step, solvent was

evaporated, and the crude product was washed with water 3 times (3x5mL) with water and dried over magnesium sulfate. The product solution in dichloromethane was precipitated into cold diethyl ether, and the pure product was obtained as a white solid. (Yield: 1.30 g, 65%)

^{1}H NMR (500 MHz, $CDCl_3$): δ_H (ppm) 3.65 (130H, t, **a**), 3.39 (3H, s, **b**)

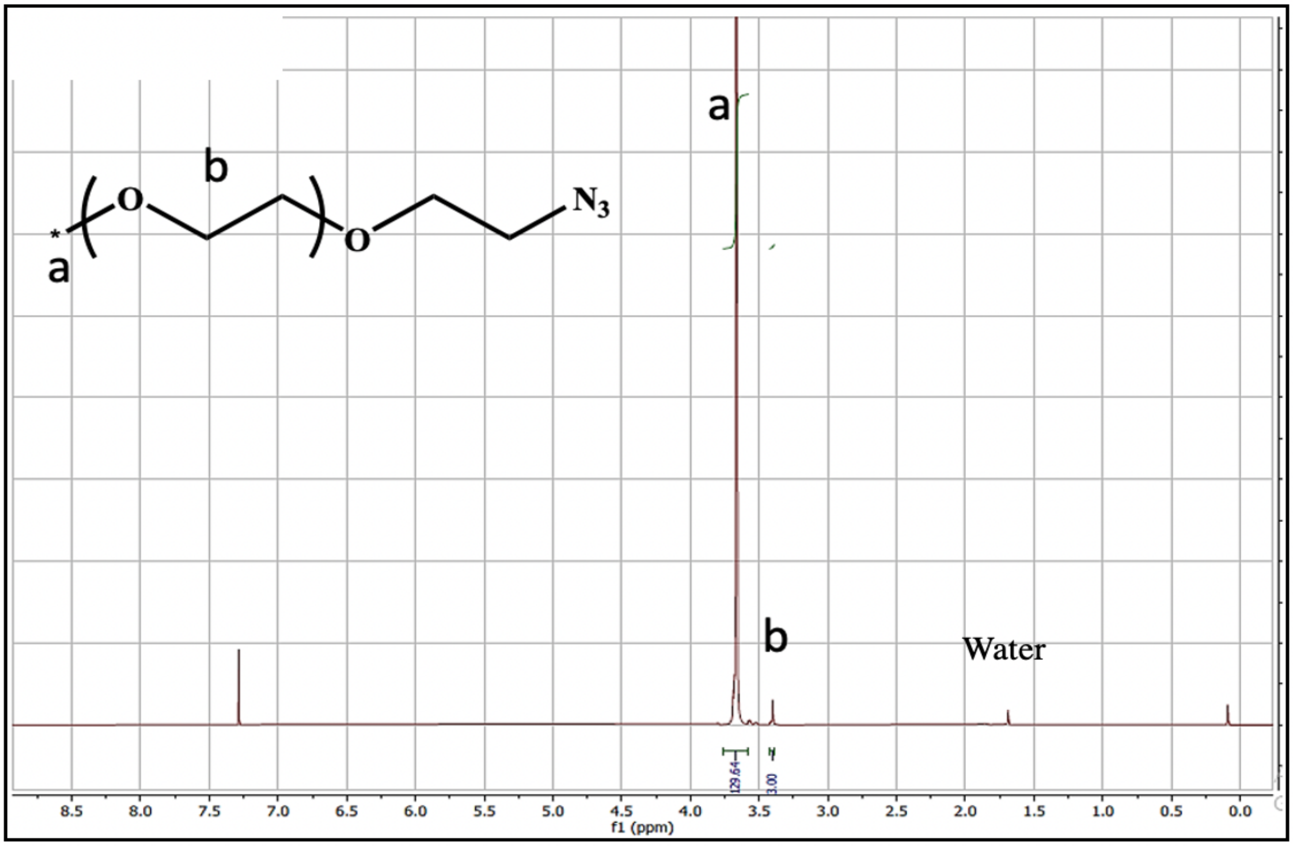

Figure S8 1HNMR for P4

Synthesis of PCL-(PEG)₂ miktoarm Polymer (P5):

Compounds **4** (0.60 g, 0.30 mmol) and **2** (0.35 g, 0.14 mmol) were dissolved in dry THF, followed by the addition of copper (I) bromide (65.0 mg, 0.450 mmol) and PMDETA (0.09 mL, 0.5 mmol) into the reaction mixture. The latter was stirred at 42°C during 48-hour period under nitrogen. After reaction completion, THF was removed, and the crude product was washed with 0.1 M EDTA solution for the copper removal. The crude product was dissolved in THF and transferred into the Spectra/ Por3 dialysis membrane (Standard RC, 3.5 kDa MWCO) and dialyzed against 1: 4 ratio of water/ THF mixture over 2 days, and the dialysis media was changed once a day. Afterwards, solvent was

removed from the mixture, and the pure product was dissolved in DCM and precipitated into the cold ether as a solid powder. (Yield: 0.40 g, 40%)

^{1}H NMR (500 MHz, $CDCl_3$): δ_H (ppm) 7.85 (2H, s, **a**), 6.62 (1H, t, **b**), 6.61 (2H, d, **c**), 5.19 (4H, s, **d**), 5.05 (2H, s, **e**), 4.56 (4H, t, f), 4.06 (65H, t, **g**), 3.90 (4H, t, **h**), 3.65 (351H, t, **i**), 3.39 (6H, s, **j**), 2.32 (65H, m, **k**),1.67 (147H, m, **l**), 1.40 (67H, m, **m**).

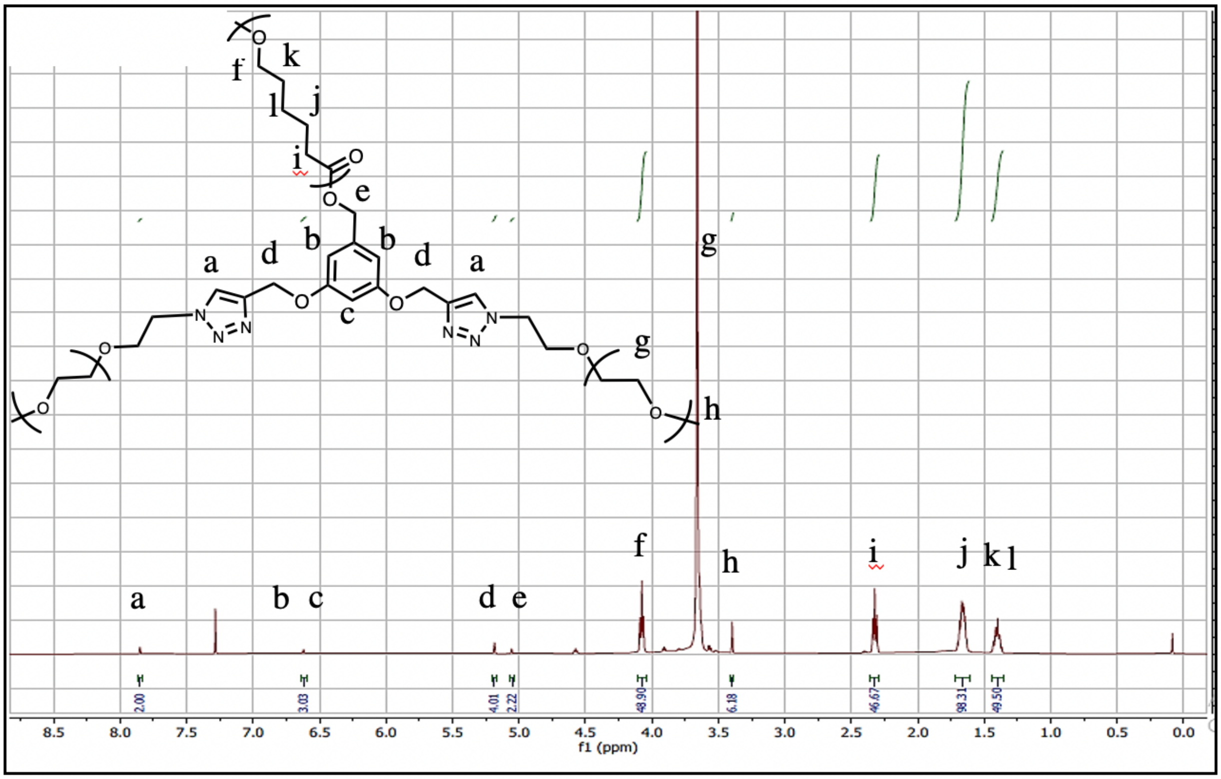

Figure S9 1HNMR for P5

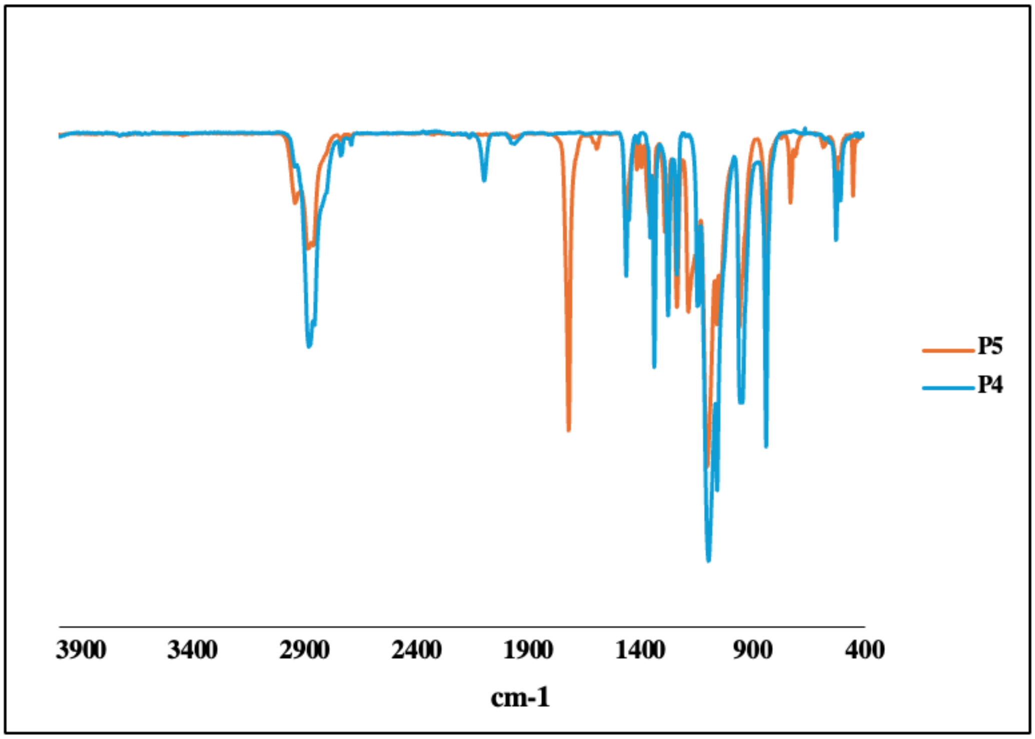

Figure S10 IR spectra for P4 and P5; The peak at 2100 cm^{-1}indicates the presence of azide group. The azide peak disappeared in P5 due to the successful click chemistry

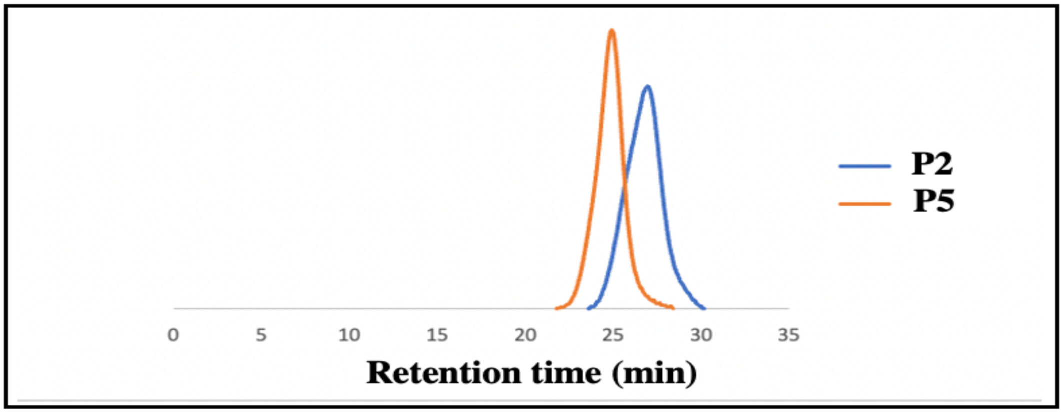

Figure S11 GPC analysis for P2 and P5

Table S1Cryoprotectant concentration assessment: The relative diameter of blank Lyo nanoparticles compared to original nanoparticles and poly dispersity index of Lyo nanoparticles using PEG_{2k} and PEG_{5k} with different concentrations

Cryoprotectant	Relative Diameter (Lyo/Original)	Polydispersity Index
**Original NP**	**1**	**0.25 ± 0.03**
**No cryoprotectant**	**43.61 ± 1.63**	**0.19 ± 0.18**
**PEG_{2k}-1%**	**3.02 ± 0.05**	**0.23 ± 0.05**
**PEG_{2k}-5%**	**9.01 ± 2.09**	**0.29 ± 0.02**
**PEG_{2k}-10%**	**27.75 ± 5.50**	**0.42 ± 0.27**
**PEG_{5k}-1%**	**6.32 ± 0.09**	**0.26 ± 0.03**
**PEG_{5k}-5%**	**16.76 ± 12.84**	**0.27 ± 0.01**

Table S2 Systematic Evaluation of PEG_{2k} and PEG_{5k} at 1% w/v: Hydrodynamic diameter and polydispersity index of blank (on the left) and curcumin-loaded nanoparticles (on the right) as Original; Non-lyophilized (NLyo); Lyophilized (Lyo)

	**Blank**		**Curcumin-loaded**	
**Cryoprotectant**	**Diameter (nm)**	**Polydispersity Index**	**Diameter (nm)**	**Polydispersity Index**
**Original NP**	**43.22 ± 0.95**	**0.25**	**37.74 ± 2.98**	**0.24**
**NLyo-PEG_{2k}-1%**	**44.31 ± 2.27**	**0.25**	**34.84 ± 1.26**	**0.24**
**Lyo-PEG_{2k}-1%**	**130.8 ± 1.82**	**0.23**	**130.3 ± 5.65**	**0.28**
**NLyo-PEG_{5k}-1%**	**54.09 ± 4.38**	**0.24**	**47.27 ± 1.47**	**0.26**
**Lyo-PEG_{5k}-1%**	**273.42 ± 3.13**	**0.25**	**238.56 ± 2.31**	**0.25**

Table S3 Alternative cryoprotectant evaluation: Relative diameter of blank Lyo nanoparticles compared to original nanoparticles and poly dispersity index of redispersed nanoparticles supplemented with 1% w/v of cryoprotectants

Cryoprotectant	Relative Diameter ratio (Lyo/Original)	Poly dispersity Index
**Original NP**	**1**	**0.25 ± 0.03**
**PEG_{2k}**	**3.02± 0.05**	**0.23 ± 0.05**
**Mannitol**	**10.67± 7.34**	**0.27± 0.08**
**Glucose**	**63.22± 16.39**	**0.47***
**Sucrose**	**6.17± 2.17**	**0.19± 0.06**
**Trehalose**	**5.27± 1.29**	**0.24± 0.09**

*A large variation was noted in the polydispersity index.
